# Crystal Structures of Bovine CD1d Reveal Altered αGalCer Presentation and a Restricted A’ Pocket Unable to Bind Long-Chain Glycolipids

**DOI:** 10.1371/journal.pone.0047989

**Published:** 2012-10-23

**Authors:** Jing Wang, Joren Guillaume, Nora Pauwels, Serge Van Calenbergh, Ildiko Van Rhijn, Dirk M. Zajonc

**Affiliations:** 1 Division of Cell Biology, La Jolla Institute for Allergy and Immunology, La Jolla, California, United States of America; 2 Laboratory for Medicinal Chemistry (FFW), Ghent University, Ghent, Belgium; 3 Department of Infectious Diseases and Immunology, Faculty of Veterinary Medicine, Utrecht University, Utrecht, The Netherlands; 4 Division of Rheumatology, Immunology and Allergy, Brigham and Women's Hospital, Harvard Medical School, Boston, Massachusetts, United States of America; Johns Hopkins University, United States of America

## Abstract

NKT cells play important roles in immune surveillance. They rapidly respond to pathogens by detecting microbial glycolipids when presented by the non-classical MHC I homolog CD1d. Previously, ruminants were considered to lack NKT cells due to the lack of a functional CD1D gene. However, recent data suggest that cattle express CD1d with unknown function. In an attempt to characterize the function of bovine CD1d, we assessed the lipid binding properties of recombinant *Bos taurus* CD1d (boCD1d) in vitro. BoCD1d is able to bind glycosphingolipids (GSLs) with fatty acid chain lengths of C_18_, while GSLs with fatty acids of C_24_ do not bind. Crystal structures of boCD1d bound to a short-chain C_12_-di-sulfatide antigen, as well as short-chain C_16_-αGalCer revealed that the Á pocket of boCD1d is restricted in size compared to that of both mouse and human CD1d, explaining the inability of long chain GSL’s to bind to boCD1d. Moreover, while di-sulfatide is presented similarly compared to the presentation of sulfatide by mouse CD1d, αGalCer is presented differently at the cell surface, due to an amino acid Asp151Asn substitution that results in loss of intimate contacts between the αGalCer headgroup and CD1d. The altered αGalCer presentation by boCD1d also explains its lack of cross-activation of mouse iNKT cells and raises the interesting question of the nature and function of bovine lipid-reactive T cells.

## Introduction

CD1 is a family of antigen-presenting molecules that is structurally related to major histocompatibility class I (MHC I) molecules, but binds and presents lipids, glycolipids and lipopeptides, rather than peptides [1]. In human, five isotypes exist (CD1a-e). While CD1a-d are differentially expressed at the cell surface of various cell types, including immature thymocytes, immature and mature dendritic cells and B cells, CD1e is exclusively associated with lysosomes where it assists in antigen processing and loading [2], [3]. CD1 is formed by a heavy chain that non-covalently associates with beta-2-microglobulin “light” chain (β2m) [4]. A transmembrane domain at the C-terminus of the heavy chain anchors CD1 to the cell surface and a short intracellular cytoplasmic tail usually contains amino acid motifs responsible for the recruitment of adapter proteins and subsequent intracellular trafficking of CD1 [5]. The heavy chain has three extracellular domains (α1, α2 and α3). While the α3 domain non-covalently associates with β2m, the α1 and α2 ectodomains form the antigen-binding groove that accommodates the lipid anchor of the antigen, while the polar headgroup, usually a carbohydrate in case of a glycolipid, is exposed at the surface for recognition by a T cell receptor (TCR) [4]. The overall architecture of all the CD1 binding grooves is conserved and allows the binding of lipid antigens that are similar in structure, e.g phosphoglycerolipids that were acquired in the ER during folding of the nascent CD1 heavy chain. However, differences in the size, shape and number of the binding groove forming pockets (A, C, F, T) give rise to distinct binding properties for unusually long or substituted lipid backbones [1]. As such, the CD1b isotype can bind antigens with sizes that exceed the binding capabilities of other CD1 isotypes, such as found in myolic acids (C_55–80_), while other binding grooves are more restricted in size, such as CD1a that can only contain alkyl chains in the A’ pocket not exceeding C_18_ [6]. Therefore, it is likely that each CD1 isotype has evolved to bind a particular class of lipid antigens, notwithstanding that they also have a overlapping binding specificities for the major antigens found in all organisms, such as diacylglycerolipids.

While humans express all 5 CD1 isotypes (CD1a-e), muroid rodents only express CD1d, while guinea pigs express multiple isoforms of CD1b and CD1c [7]. Non-primate mammals also lack some isotypes and/or express multiple variants of certain isotypes [8], [9]. In particular, cattle possess three functional *CD1B* genes, called *CD1B1*, *CD1B3* and *CD1B5* that differ in their sequence and shape of the CD1b binding groove and in their cytoplasmic tail [8], [10]. It, therefore, appears that certain species functionally compensate for the lack of one CD1 isotpye by either expressing several other isotypes or by using a multifunctional isotype, such as murine CD1d.

CD1d is the restricting element for Natural Killer T (NKT) cells that have been widely studied in both humans and mice [11]. There are two major classes of NKT cells. Type I NKT cells are characterized by the conserved TCR α-chain rearrangement (Vα14Jα18 in mouse and Vα24Jα18 in humans) and respond to the model antigen αGalCer, whereas Type II NKT cells use an oligoclonal TCR α and TCR β chain repertoire and do not share a common antigen [12]. However, many of the type II NKT cells respond to the self-antigen sulfatide [13], [14]. Both Type I and II NKT cells rapidly respond to antigen stimulation and produce both pro- and anti-inflammatory effector cytokines, such as IL-4 and IFN-y.

It was previously reported that cattle do not express functional CD1d due to the lack of a start codon [8]. However, a recent study verified cell surface expression of full-length bovine (bo) CD1d in cattle [15] and cattle also have the necessary TCR genes to express NKT cells [16]. This raises the question as to what the antigen is for boCD1d and whether cattle have functional NKT cells, similar to humans and mice or use a different CD1d-restricted T cell population.

In an attempt to shed light onto the ability of boCD1d to function as an antigen presenting molecule, we have expressed recombinant boCD1d and used the two well-studied human and murine NKT cell ligands, sulfatide and α-galactosylceramide (αGalCer) to characterize their presentation by boCD1d.

## Materials and Methods

### Oligonucleotides

boCD1d-down: 5′-gactgtcgacatgcggtacctaccatggctgttgctgtgg gcattcctacaggtctggggacaatctccagccccgcaaacgcc-3′.

boCD1ab-up: 5′-gaggatccttagtgatggtgatggtgatgccagtagaggatgatgtcctgg-3′.

b2Mfus-down: 5′-atacaattgatccagcgtcctccaaagattc-3′.

b2Mfus-up: 5′-ttgcggccgcgatgatcctcctccgcttcctgatcctccgcttcctcctcctcccatgtctcgatcccacttaac-3′.

boCD1dfus-f: 5′-aagcggccgcaagccccgcaaacgcctttc-3′.

boCD1dfus-r: 5′-ataggatccgcgcggcaccagtccccagtacaggatgatgtcctg-3′.

mCD1d-D153N-f: 5′-tcaaagtgctcaacgctaatcaagggacaagtgca-3′.

boCD1d-N151D: 5′-caaggtgctcaatcaggaccaagggaccaagga-3′.

boCD1d-QN-AD: 5′-ggtcatcaaggtgctcaatgcggaccaagggaccaagg-3′.

### Cloning, Expression and Purification of Bovine CD1d

The boCD1d ectodomain, in which the endogenous boCD1d leader sequence was replaced with that of boCD1b3 was amplified by PCR using the primers boCD1d-down and boCD1ab-up. The resulting PCR fragment was restricted with *Sal*I and *Sph*I and ligated into the boCD1b3 containing dual-promotor baculovirus transfer vector pBACp10pH by replacing the boCD1b3 fragment [10]. The resulting plasmid contained both boCD1d with a C-terminal hexa-histidine tag, as well as bovine β2-microglobulin (boβ2M). This construct was used for crystallization.

For functional studies boCD1d was also expressed as a single chain Fc-fusion protein, in which boβ2M (without leader sequence, amplified using the primers b2M-fus-down and –up) was connected to the N-terminus of the boCD1d heavy chain through a (G_4_S)_3_ amino acid linker contained within the primer b2Mfus-up, while the C-terminus of the heavy chain (amplified with primers boCD1dfus-f and-r) was further fused to the Fc-region of human IgG1 that had previously been cloned into the baculovirus transfer vector pAcGP67A (BD Biosciences). The recombinant soluble bovine CD1d-β2m heterodimeric molecule without Fc tag was produced to homogeneity using the baculovirus expression system as previously described for mouse CD1d [17] and stored at −80**°**C for lipid loading studies and crystallization. The single chain boCD1d-Fc fusion protein was purified from a 5 L culture of *Spodoptera frugiperda* (SF9) insect cells (EMD Biosciences) that had been infected with the corresponding recombinant baculovirus at an MOI ∼3 for 72h at 27.4**°**C. Briefly, SF9 cells were removed from the 5 L culture by gentle centrifugation (1,000×g, 10 min) and the supernatant concentrated to 300 mL using a tangential flow through filtration device (Pelllicon 2, Millipore) while exchanging the buffer to PBS. The boCD1d-Fc fusion protein containing supernatant was passed through a protein A column (GE Healthcare, Hi Trap™ rProtein A), washed with 3 column volumes of PBS and eluted with 20 mM glycine, 50 mM NaCl, pH 3.0. Fractions of 0.7 ml were collected into tubes prefilled with 0.3 ml 1 M Tris pH 8.5 for neutralization. Fractions containing boCD1d-Fc were analyzed by 4–20% SDS-PAGE, pooled and buffer exchanged against PBS, using centrifugal filtration devices (Amicon, 30 kDa molecular weight cut-off).

### Synthesis of C_16_-αGalCer

**Figure 1:**
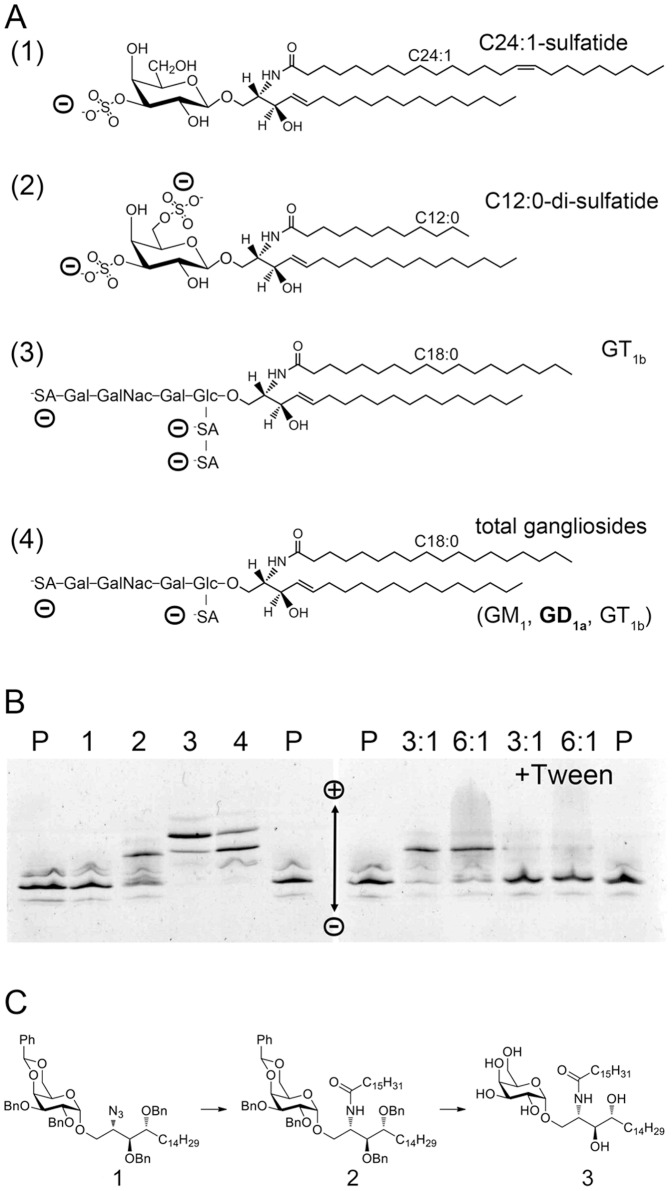
Gycolipid binding analysis of boCD1d assessed by native IEF . (A) Chemical structures of the utilized lipids bovine brain sulfatide (major species C_24∶1_-sulfatide, 1), C_12∶0_-di-sulfatide (2), GT_1b_ (3) and total gangliosides (GM_1_, GD_1a_, GT_1b_), with GD1a represented as one major species (4). The negative charges of the glycolipids are indicated as (-) and correlate with the observed gel shift upon binding to boCD1d represented as illustrated in (B). (B) native IEF gel illustrating loading of boCD1d with different lipids (left panel), numbered as in (A). Right panel assesses loading efficiency of C_12∶0_-di-sulfatide (1) with either 3× (3∶1) or 6× (6∶1) molar excess of lipid, loaded in the presence (+Tween) or absence of 0.05% Tween 20. P indicates boCD1b protein used for loading. Length of the fatty acid is indicated for each ligand and represents the major fatty acid of the natural glycolipids sulfatide (C_24∶1_), total gangliosides (C_18∶0_) and GT_1b_ (C_18∶0_). Of note, only 5% of the fatty acids are shorter than C_20∶0_, while 7% are shorter than C_22∶0_. (C) Synthesis of C16∶0-αGalCer [(2R,3S,4S)-1-O-(α-D-galactopyranosyl)-2-palmitoylamino-1,3,4-octadecanetriol, **3**] starting from a known azide **1,** via the intermediate precursor **2.**

**(2R,3S,4S)-3,4-di-O-benzyl-1-O-(2,3-di-O-benzyl-4,6-O-benzylidene-α-D-galactopyranosyl)-2-palmitoylamino-1,3,4-octadecanetriol.** To a solution of known azide (1, Figure 1c) (150 mg, 0.16 mmol) in 1.6 ml tetrahydrofuran (THF) at room temperature, a 1M solution of PMe_3_ in THF (1.6 ml, 1.6 mmol) was added dropwise. After stirring for 2.5 h at room temperature, 2.9 ml of a 1M NaOH solution was added and the reaction mixture was allowed to stir for an additional 2h. The reaction was extracted with EtOAc and the combined organic layer was washed with a saturated aqueous NaCl solution (brine), dried over Na_2_SO_4_, filtered, and evaporated to afford the crude amine. A mixture of the crude amine, 1-ethyl-3-(3-dimethyllaminopropyl)carbodiimide hydrochloride (EDC, 60 mg, 0.31 mmol) and palmitic acid (80 mg, 0.31 mmol) in 2,4 ml dichloromethane (DCM) was stirred overnight at room temperature. The reaction mixture was diluted with DCM, washed with H_2_O (2×5 ml) and brine (1×5 ml), dried over Na_2_SO_4_, filtered, and evaporated to dryness. Purification of the residue by flash chromatography (silica gel, hexane:EtOAc 7∶3) afforded 131 mg of compound 2 (Figure 1c) (72% yield) as a white solid. Exact mass (ESI-MS) for C_75_H_108_NO_9_ [M+H]^+^ found, 1166.8021 calculated, 1166.8024.

**(2R,3S,4S)-1-O-(α-D-galactopyranosyl)-2-palmitoylamino-1,3,4-octadecanetriol (C_16∶0_-αGalCer).** A solution of **2** (130 mg, 0.11 mmol) in 4 ml EtOH/CH_3_Cl 3∶1 was hydrogenated for 1 h under atmospheric pressure in the presence of Pd black (20 mg). The solution was diluted with 5 ml of pyridine, filtered through celite and evaporated to dryness. The residue was purified by flash chromatography (silica gel, DCM:MeOH 9∶1) to give 46 mg of compound **3** (58% yield) as a pale yellow solid.

^1^H NMR (300 MHz, pyridine-*d*_5_) δ ppm 0.76–1.10 (m, 6 H, CH_3_ terminal) 1.15–1.48 (m, 46 H, CH_2_) 1.61–1.99 (m, 6 H, CH_2_) 2.29 (br s, 1 H, OH) 2.46 (t, J = 7.4 Hz, 2 H, H-2′) 4.25 - 4.49 (m, 6 H, H-3″, H-4″, H-5″, H-3, H-4 and H-6″) 4.53 (t, J = 6.2 Hz, 1 H, H-3″) 4.57 (d, J = 3.1 Hz, 1 H, Ha-1) 4.63–4.73 (m, 2H, Hb-1 and H-2″) 5.24–5.34 (m, 1 H, H-2) 5.59 (d, J = 3.7 Hz, 1 H, H-1″) 8.48 (d, J = 8.5 Hz, 1 H, NH(CO)).

^13^C NMR (75 MHz, pyridine-*d*_5_) δ ppm 11.52, 14.60, 14.69, 23.35, 23.62, 24.47, 26.80, 26.92, 29.57, 30.03, 30.16, 30.22, 30.27, 30.34, 30.40, 30.41, 30.44, 30.56, 30.78, 31.08, 32.54, 34.78, 37.21, 39.48, 51.83, 63.08, 68.58, 69.06, 70.71, 71.42, 72.02, 72.91, 73.45, 77.15, 101.94, 173.63.

Exact mass (ESI-MS) for C_40_H_79_NNaO_9_ [M+Na]^+^ found, 740.5665 calculated, 740.5653.

### *In vitro* Loading of Lipid Antigens

Brain porcine sulfatide extract, C_12_-di-sulfatide and total brain gangliosides were purchased from Avanti Polar Lipids Inc. and purified trisialoganglioside G_T1b_ from bovine brain was obtained from Sigma Aldrich (minimum purity 96%). Lipids were dissolved at 1 mg/ml in DMSO to assess *in vitro* loading of antigens by boCD1d. Aliquots of 5 µg of purified boCD1b3 at a concentration of 10 µM were loaded overnight at room temperature in the presence of 30 or 60 µM of each ligand (in DMSO, final concentration of DMSO 3–5%) with or without Tween 20 at a final concentration of 0.05%. Loading was performed in the presence of 100 mM TrisHCl pH 7. The binding of charged lipids was assessed by isoelectric focusing on a PhastGel IEF 3–9 using a PhastSystem (GE Healthcare) followed by staining with Coomassie dye.

### Protein Crystallization

Crystallization was performed in a 96-well format by a nanoliter dispensing liquid handling robot (Phoenix, Art Robbins Ltd.) using commercially available crystallization screens (PEG/Ion I & II, Wizard I & III, Hampton Research and Emerald Biosciences). Sitting drops of 100 nl precipitant were overlayed with 100 nl of protein solution (6.7 mg/ml in 10 mM Hepes, 30 mM NaCl, pH 7.5). A single diffraction quality crystal of boCD1d-C_16_-αGalCer was obtained from the PEG/Ion screen (20% polyethylene glycol 4000, 0.2 M potassium sulfate) and used for subsequent data collection. Promising conditions for the boCD1d-di-SLF complex were optimized manually and diffraction quality crystals were grown by sitting drop vapor diffusion at 22**°**C, mixing 0.5 µl of protein at 6.7 mg/ml with 0.5 µl of precipitant (20% polyethylene glycol 3350, 0.2 M sodium formate).

### Structure Determination and Refinement

Crystals were flash-cooled at 100K in crystallization solution containing 20% glycerol. Diffraction data of the bo-CD1d-di-SLF crystal were collected at beamline 5.0.3 of the Advanced Lightsource (ALS, Berkeley, CA) and data of the boCD1d-C_16_-αGalCer crystal were collected at beamline 11.1 at the Stanford Synchrotron Radiation Lightsource (SSRL, Stanford, CA) and processed with the HKL2000 software to a resolution of 2.9 Å and 2.4 Å, respectively [18]. Molecular replacement was performed using Phaser [19], and the homology model of boCD1d generated from the boCD1db3 structure (PDB code 3L9R) using the Swiss Model server [20]. The asymmetric unit (ASU) of the boCD1d-di-SLF crystal contained two CD1d-β2m heterodimers, while the ASU of boCD1d-C_16_-αGalCer contained one heterodimer. The initial phases derived from the molecular replacement solution were refined using maximum-likelihood restrained refinement in Refmac [21]. Refinement was intercalated with rounds of manual model building into 2F_o_-F_c_ and F_o_-F_c_ maps in Coot [22]. Tight NCS constraints between the two boCD1d-di-SLF molecules present in the ASU were imposed and maintained throughout refinement. TLS refinement was performed at later stages for the boCD1d-C_16_-αGalCer structure in Refmac [21], [23]. Three TLS groups per boCD1d-β2M complex were defined, containing the α1, α2 domains and the C_16_-αGalCer ligand (group 1), the α3 domain (group 2) and the β2m chain (group 3). A set of ∼ 1000 reflections (4%) were set aside for the calculation of R_free_ to monitor refinement progress. The quality of the final model was assessed with Molprobity [24]. Data collection and refinement statistics are presented in Table 1.

**Table 1:** Data collection and refinement statistics.

	boCD1d-di-SLF	boCD1d-C_16_-αGalCer	
**Data collection**			
Space group	P 4_2_	P 2_1_2_1_2_1_	
Cell dimensions			
*a, b, c,* (Å)	168.55, 168.55, 41.53	55.79, 74.48, 122.92	
α = β = γ (°)	90.00	90.00	
Resolution range (Å)	50.0–2.90 [3.00–2.90]	40.0–2.40 [2.46–2.40]	
Completeness (%)	99.3 [98.4]	97.4 [98.2]	
Number of unique reflections	27,330	20,240	
Redundancy	3.9	4.1	
R_sym_ (%)	11.4 [58.6]	6.7 [19.9]	
I/σ^a^	17.9 [3.0]	35.0 [4.9]	
**Refinement statistics**			
Number of reflections (F>0)	26,175	19,328	
Maximum resolution (Å)	2.86	2.40	
R_cryst_ (%)	22.2 [26.5]	22.1 [40.1]	
R_free_ (%)	29.1 [35.3]	27.6 [39.0]	
**Number of atoms**	6,216	3,070	
Protein	5,925	2,947	
Glycolipid	106	50	
N-linked carbohydrate	156	42	
Solvent molecules (waters/sulfate)	29/0	26/5	
**Ramachandran statistics (%)**			
Favored	96.8	95.6	
Outliers	0.28	0.0	
**R.m.s.d. from ideal geometry**			
Bond length (Å)	0.013	0.011	
Bond angles (°)	1.52	1.40	
**Average B values (Å^2^)**			
Protein	44.9	61.0	
Glycolipid	70.7	82.8	
Water molecules	35.4	52.0	
Carbohydrates	77.4	92.0	

Numbers in parentheses refer to the highest resolution shell.

### Accession Numbers

Structure factors and coordinates for the boCD1d-C_16_-αGalCer and boCD1d-di-SLF structures have been deposited into the PDB database (http://www.rcsb.org/) with accession codes 4F7E and 4F7C, respectively.

### Cell Free Antigen Presentation Assay

The cell-free Ag presentation assay for stimulation of mouse iNKT cell hybridomas by recombinant mouse, human and bovine CD1d was carried out according to published protocols [25], [26] with the following modifications. Briefly, 1 µg soluble hCD1d, mCD1d, mCD1d Asp153Asn, boCD1d, boCD1d Asn151Asp and boCD1d Asn151Asp/Gln150Ala proteins were coated in 96-well flat-bottom plates at 37 °C for 1 h. Plates were blocked for 1 h with PBS containing 10% FBS and 100µl of glycolipids αGalCer (KRN7000) or C_16_-αGalCer (dissolved in 0.05% Tween 20 and 0.9%NaCl) were added to each well at various concentrations and incubated for 20 h at 37 °C. After washing, 5x10^4^ hybridoma cells in 200 µl complete media were added to each well and incubated at 37 °C for 16 h in a CO2 incubator. IL-2 release in the supernatant (100 µl) was measured in a sandwich ELISA, as previously described [25], [26].

### Figure Preparation

Lipid structures were prepared in ChemDraw (CambridgeSoft), all molecular representations were prepared using PyMol (Schroedinger). Protein cavities were prepared using the fpocket webserver (http://bioserv.rpbs.univ-paris-diderot.fr/fpocket/) [27] and visualized in PyMol. Lipid binding groove volumes were calculated using the CASTp server (http://sts-fw.bioengr.uic.edu/castp/) [28].

## Results

### Bovine CD1d can Bind and Present Lipid Antigens *in vitro*

Bovine CD1d (boCD1d) was previously considered a pseudogene but is translated and expressed at the cell surface, when transfected into 293 T cells using the native CD1d leader peptide [8], [15]. To investigate whether boCD1d can bind glycolipids *in vitro*, we have recombinantly expressed the ectodomain of boCD1d in insect cells and assessed the glycolipid binding properties of boCD1d using native isoelectric focusing gel electrophoresis. Several charged ligands (Figure 1a) were incubated with boCD1d and successful loading was visualized by native IEF gel (Figure 1b). Loading of a negatively charged lipid resulted in a gel shift toward the cathode, caused by the charge difference of the CD1d-lipid complex. While boCD1d bound C_12∶0_-di-sulfatide, total gangliosides and GT1b, it failed to bind bovine brain sulfatides (Figure 1B). Besides differences in the headgroups, which generally do not affect the ability of the antigen to bind to CD1, the glycolipids only differ in the length and saturation of the fatty acid, whereas the long chain base (sphingosine) is identical (Figure 1A). While gangliosides and GT1b contain predominantly C_18∶0_ fatty acids, di-sulfatide is significantly shorter (C_12∶0_) and the fatty acids of bovine brain sulfatides typically range from 22 to 24 carbons in length. This indicated that bovine brain sulfatides could potentially exceed the chain length for binding to boCD1d. For comparison, the lipid binding groove of mouse and human CD1d can accommodate fatty acids with up to 26 carbons in the A’ pocket [17], [29], [30]. Next, we tested whether increasing the molar ratio of C_12∶0_-di-sulfatide and/or including 0.05% Tween 20 detergent during lipid loading could increase the loading efficiency (Figure 1B, right panel). Unexpectedly, increasing lipid excess did not increase loading efficiency, which already appeared optimal, while in contrast, the addition of Tween 20 prevented C_12∶0_-di-sulfatide binding altogether. This observation could be specific for C_12∶0_-di-sulfatide and indicates that this lipid is predominantly trapped in mixed lipid/detergent micelles, therefore being unavailable for loading to boCD1d. BoCD1d ran as a triplet on the IEF gel, with the majority of the protein migrating as the center band. Upon addition of Tween 20, however, the middle band remains unchanged, while the fainter top and bottom bands disappeared, potentially indicating extraction of single positively and negatively charged endogenous lipids that had been acquired during protein expression and folding in the ER of insect cells.

### Crystal Structure of boCD1d

**Figure 2:**
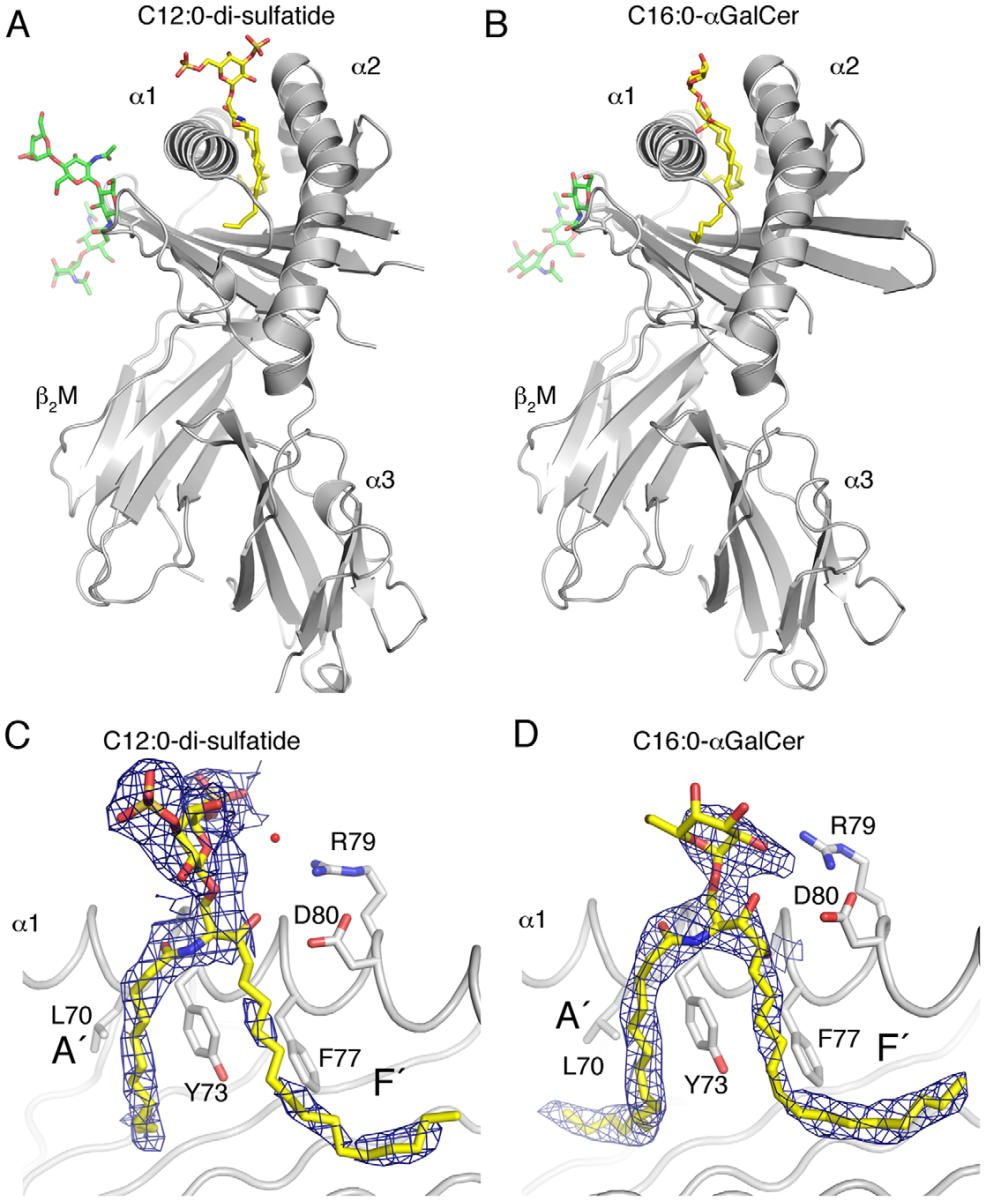
Overview of the boCD1d-ligand structures. CD1 heavy chain with α1, α2 and α3 domains indicated and β2m are shown in grey. The bound C_12∶0_-di-sufatide (A) and the C16∶0- αGalCer (B) ligands are shown as yellow sticks within the boCD1d binding helices. *N*-linked oligosaccharides are shown in green. Binding orientation within the CD1d binding groove and 2FoFc electron density (contoured at 1σand shown within a 2 Å radius around the glycolipid) is shown as a blue mesh for C_12∶0_-di-sufatide (C) and C_16∶0_-αGalCer (D). A water molecule is shown as a red sphere in (C). The two major binding pockets A’ and F’ are indicated as such.

To address the question of why boCD1d does not bind bovine brain sulfatides to a measurable degree, we crystallized boCD1d in complex with synthetic C_12∶0_-di-sulfatide (di-SLF) and determined the crystal structure to a resolution of 2.9 Å (Table 1 and Figure 2A). The obtained structural information encouraged us to synthesize a shorter form of αGalCer with a C_16∶0_ instead of the typical C_26∶0_ fatty acid (Figure 1C), which we also crystallized bound to boCD1d. The crystal structure of the boCD1d-C_16∶0_-αGalCer complex was determined to a resolution of 2.4 Å (Table 1 and Figure 2B). The asymmetric unit of the boCD1d-di-SLF crystal contained two separate boCD1d-boβ2M-di-SLF complexes that are very similar, especially in the presentation of the bound glycolipid. Therefore, we will present structural information for only one complex. The overall structure of boCD1d is very similar to that of other CD1 or MHC I molecules, in which the boCD1d heavy chain is organized in 3 domains, α1 and α2 that form the central binding groove and α3, which non-covalently associates with boβ2M (Figure 2) [1]. The glycolipids are bound in the hydrophobic binding groove that can further be divided into the two main pockets, A and F. While the A pocket binds the acyl chain of each glycolipid, the F pocket binds the sphingosine, leaving the structurally distinct carbohydrate headgroups exposed above the boCD1d binding groove for immune recognition by cognate T cells (Figure 2). Unambiguous electron density is observed for the galactosyl-di-sulfate headgroup and the C_12∶0_ fatty acid of di-sulfatide, while in case of C_16∶0_ -αGalCer, electron density is well defined for the entire lipid backbone but less well ordered for the galactose headgroup (Figure 2C, D and Figure S1). This is in contrast to the well ordered electron density of the αGalCer headgroup, when presented by either mouse or human CD1d, indicating that the interaction between the galactose headgroup and boCD1d is not conserved with that of human or mouse CD1d [29], [30]. Details about the headgroup presentation by boCD1d will be discussed later.

The boCD1d α1-α2 superdomain (residues 1–180) shares 60% sequence identity (BLAST search) with human CD1d and 55% with mouse CD1d. Superposition of the entire boCD1d heavy chain from the di-sulfatide complex with that of huCD1d and mCD1d yielded rmsd values of 0.92 Å (PDB 1ZT4) and 1.13 Å (PDB 1Z5L), respectively, indicating a very similar topology of the boCD1d molecules. Interestingly, superposition of both boCD1d crystal structures resulted an rmsd of 0.62 Å, suggesting a degree of flexibility of the binding groove when it accommodates different glycolipids.

Cross-species conserved *N*-linked oligosaccharides are observed at residues Asn20 and 42, while the third conserved site on the α2-helix of CD1d (N163 in human, N165 in mouse) is lost in boCD1d (H163) (Figure 2).

### boCD1d has a Restricted A’ pocket

**Figure 3:**
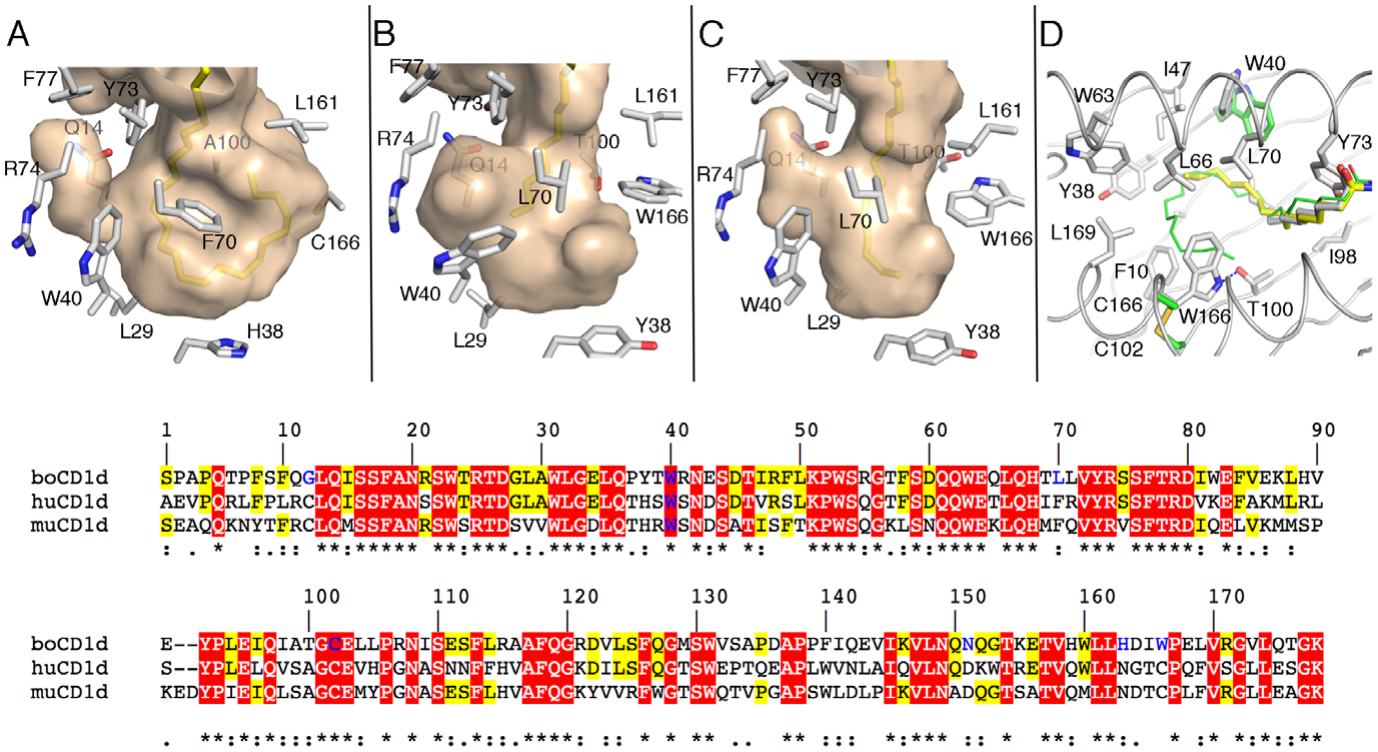
Architecture of the A’ pocket of boCD1d . The A’ pocket of human CD1d with bound C_16∶0_-αGalCer (A), boCD1d with bound C_12∶0_-di-sufatide (B) and C_16∶0_-αGalCer (C) is shown as a semi-transparent molecular surface colored in mauve. CD1d residues that influence the shape of the groove are indicated as grey sticks. (D) Superposition of the A’ pocket of boCD1d and huCD1d, with C_12∶0_-di-sufatide (yellow), C_16∶0_-αGalCer (grey) and C_26∶0_-αGalCer (green, from PDB ID 1ZT4) shown. While Trp40 (W40) adopts different orientations in both boCD1d structures, which influences the shape of the small side pocket, Trp166 (W166) blocks the second half of the A’ pocket and restricts the ligand size to 16–18 carbons. Trp166 is a cysteine in both human and mouse CD1d (C102-C166 in green/yellow) that is involved in a disulfide bond with Cys102 located on the β-sheet (C). Sequence alignment of the binding groove forming residues of bovine (bo), human (hu) and murine (mu) CD1d (bottom panel). Red boxed residues are conserved in all three species, while yellow boxed residues are conserved between boCD1d and either human or mouse CD1d. Residues that represent features that are not conserved in boCD1d are colored blue (e.g. A’ pole; G12, L70, C102-C166 disulfide bond, N151 which is important for αGalCer headgroup binding and both Trp40 and Trp166 that influcne the shape of the groove).

Our *in vitro* lipid loading studies indicated that lipids with fatty acid chains lengths of 24 carbons or more, such as C_24∶1_-sulfatide or αGalCer, are unable to bind to boCD1d. As the acyl chain is bound within the A’ pocket of CD1d, we compared the A’ pockets of boCD1d containing C12∶0-di-sulfatide or C_16_-αGalCer with that of human CD1d containing full-length C_26∶0_-αGalCer (Figure 3A–D). We made two observations that have not been observed before for any CD1d molecule. Firstly, the shape and size of the boCD1d A’ pocket differs when either C_12∶0_-di-sulfatide or C_16_-αGalCer are bound indicating flexibility within the A’ pocket (Figure 3B, C). This is a result of a different conformation of the conserved residue Trp40, which can either increase the size of the small side pocket, likely induced through contacts with the terminal carbons of the C_12∶0_ acyl chain of di-sulfatide, or restrict the size when the longer C_16∶0_ fatty acid is further inserted into the Á pocket. Secondly, the conserved disulfide bond in human and mouse CD1d that anchors the α2-helix to the β-sheet platform (Cys166-Cys102) is absent in boCD1d. Here, Trp166 adopts a rare rotamer, stabilized by interaction with the side chain of Thr100, pointing inside the A’ pocket and as such blocking the remainder of the A’ pocket (Figure 3D). This results in a shorter, more restricted A’ pocket of boCD1d, which can maximally bind glycolipid antigens with an acyl chain not exceeding 18 carbons in length. The restricted A’ pocket also results in a smaller boCD1d lipid binding groove, which is 1680 Å^3^ in volume, compared to mouse (1970 Å^3^) and human (1960 Å^3^) CD1d.

### Antigen Presentation by boCD1d

**Figure 4:**
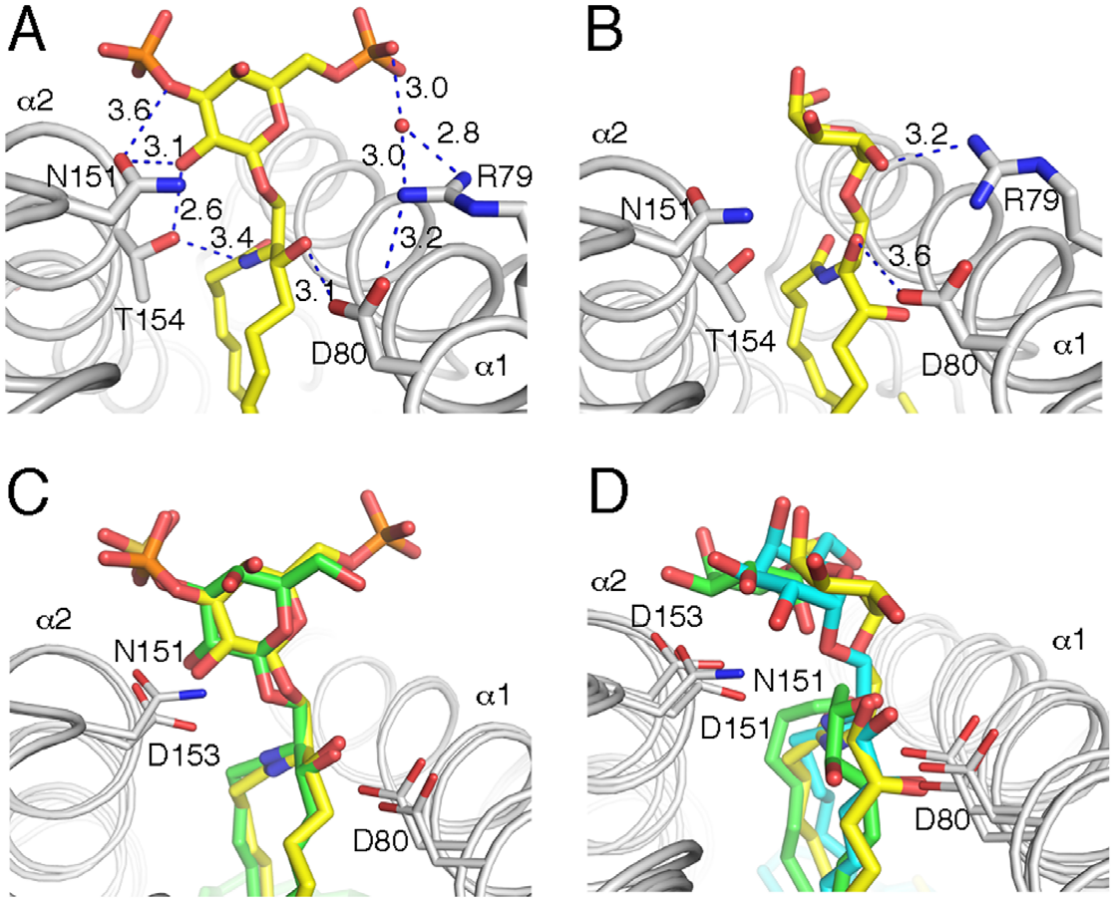
Antigen presentation by boCD1d. Presentation of the headgroups of C_12∶0_-di-sufatide (A) and C_16∶0_-αGalCer (B) above the boCD1d binding groove and comparison with mouse CD1d presented sulfatide (C) and mouse and human CD1d presented αGalCer (D). Glycolipids presented by boCD1d as yellow, mouse CD1d as green and human CD1d as cyan sticks. Note, how di-sulfatide is bound across the binding groove and in contact with both CD1d residues of the α1 and α2 helix, boCD1d–presented αGalCer looses intimate contacts with N151 and exclusively contact α1helix residues (R79 and D80), resulting in a more tilted headgroup presentation.

The di-sulfo-galactosyl headgroup of di-SLF is presented in the for β-anomeric glycolipids typical upright orientation, between the α1- and α2 helices and stabilized through an intricate network of H-bonds that involve the core residues of CD1d (Arg79, Asp80, Asn151 and Thr154), as well as the polar moieties of the di-SLF ligand (Figure 4A). The 3′-SO_4_ group contacts Asn151, while the 6′-SO_4_ group is bound through a water-mediated hydrogen (H) bond to Arg79. The ceramide lipid backbone is oriented inside the binding groove by an H-bond between Thr154 and the N-amide nitrogen, as well as by Asp80 of CD1d binding the 3″-OH group of sphingosine. A total of 6 H-bonds are formed between boCD1d and di-SLF (four with the headgroup and two with the lipid), which gives rise to ordered electron density for the headgroup (Figure 2C). Overall, the orientation of di-SLF in the boCD1d binding groove, and the presentation at the opening of the groove is conserved with the presentation of sulfatide by mCD1d (Figure 4C).

As human and mouse iNKT cells are cross-reactive in regards to αGalCer, we investigated whether the presentation of C_16∶0_-αGalCer by boCD1d is altered compared to that of either human or mouse CD1d. Indeed, the binding and presentation of C_16∶0_-αGalCer is not conserved with that of either mouse or human CD1d (Figure 4B, D). BoCD1d presents C_16∶0_-αGalCer in an untypically tilted orientation. The only polar interactions between C_16∶0_-αGalCer and boCD1d is through α1-helix residue Arg79 that interacts with the 2′-OH of galactose, as well as the contact formed between Asp80 and the 3″-OH of the phytosphingosine chain. Loss of the intimate contacts between the α-anomeric galactose and the 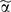 helix is likely caused by the exchange of Asp151 to Asn151. This otherwise conserved aspartate residue on the α2-helix (Asp 151 in hCD1d and Asp153 in mCD1d) is critical for the presentation of αGalCer and related antigens, by CD1d, as it binds the 2″- and 3″-OH of the galactose in human and mCD1d and substitutions to either tyrosine or alanine abrogate iNKT cell activation [31] (and data not shown).

**Figure 5:**
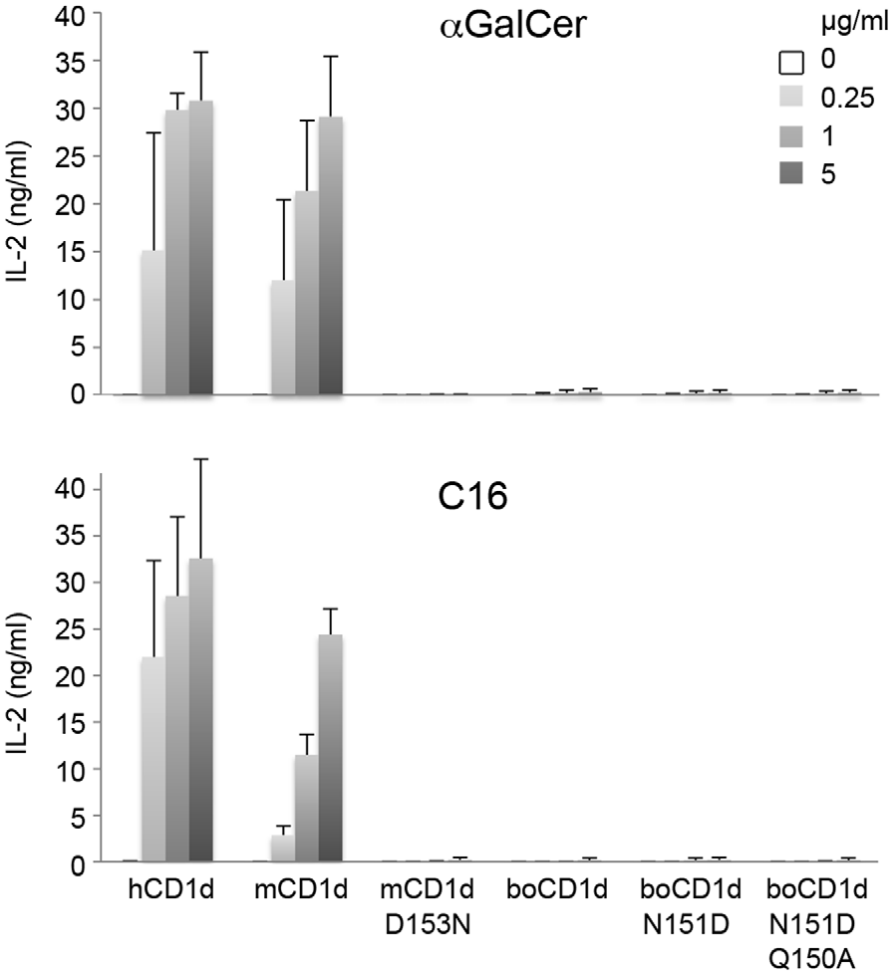
Activation of mouse NKT cells by CD1d orthologs. Activation of mouse iNKT hybridomas 1.2 by recombinant human, mouse and bovine CD1d in a CD1d-coated plate assay, using either αGalCer (top) or C_16_-αGalCer (C16, bottom). Individual amino acid mutations in CD1d are indicated. Concentrations of glycolipids used to load plate bound CD1d ranges from 0–5 µg/ml as indicated. iNKT cell activation is measured by the release of IL-2 in an ELISA assay. Experiment was performed twice, while measuring each condition in triplicate.

We next assessed, whether mutating Asp153 of mouse CD1d to Asn (as found in boCD1d) alone is sufficient for the loss of mouse iNKT cell activation and conversely, if re-constitution of Asp151 residue in boCD1d (to mimic mouse and human CD1d) can restore mouse iNKT cell activation in a cell-free antigen-presentation assay (Figure 5). We also prepared the boCD1d double mutant Asn151Asp/Gln150Ala, which further introduces a mouse CD1d residue in an area that is in close contact with the CDR3β loop of the mouse iNKT cell hybridoma 2C12 [32]. While both human and mouse CD1d can activate the two mouse iNKT cell hybridomas 2C12 as well as 1.2 by presenting either full-length αGalCer or C_16_-αGalCer, the mCD1d Asp153Asn mutant and all the bovine CD1d proteins (wt and mutants) are unable to activate both mouse iNKT cell hybridomas (Figure 5, and data not shown). Both hybridomas respond equally well to αGalCer when presented by either human or mCD1d, while 1.2 is activated better when C_16_-αGalCer is presented by human CD1d. Our data further indicates that alteration of Asp153 of mouse CD1d is not tolerated, not even when replaced with similar amino acids, such as asparagine, thereby explaining the inability of boCD1d to activate murine NKT cell. However, replacing Asn151 of boCD1d with aspartate does not reconstitute cross-species reactivity, indicating that other amino acids that differ between mouse and bovine CD1d likely are equally important for either optimal glycolipid presentation or iNKT TCR binding to CD1d. Surprisingly, however, superposition of bovine CD1d onto the structure of the mouse CD1d-αGalCer –Vα14Vβ8.2 TCR complex (PDB ID 3HE6) [33], as well as the human ortholog complex (PDB ID 3HUJ) [34], did not reveal any other obvious amino acids that are conserved between mouse and human CD1d but differ with boCD1d, and which would prevent binding of the mouse iNKT TCR onto boCD1d, raising the question as to what residues actually limit cross-species reactivity.

## Discussion

In contrast to earlier studies we could now show that cattle express CD1d protein that is able to traffic to the cell surface for antigen display [15]. In vitro binding studies indicated that glycolipids that exceed the alkyl chain length of C_18_ do not bind to boCD1d. Our structural studies using a short chain C_12_-di-sulfatide finally revealed that boCD1d has a restricted A’ pocket, which in contrast to mouse and human CD1d, can maximally bind glycolipids with alkyl chain lengths approximating C_18_. Not surprisingly, no increase in body temperature or serum cytokine levels (signs of immune activation) were observed when injecting full-length αGalCer into cattle, as it cannot be presented by boCD1d. However, human NKT cells responded weakly to both shorter versions of αGalCer (C_12_ and C_16_) [15], suggesting that the *in vivo* response against αGalCer should be re-assess using the shorter variants.

In an attempt to decipher the structural basis of glycolipid antigen-presentation by boCD1d, we determined the crystal structure of boCD1d presenting C_16_-αGalCer. We found that αGalCer is presented differently by boCD1d, due to the exchange of a single aspartate amino acid (to asparagine) that is otherwise conserved between mouse and human CD1d. The structure further reveals that asparagine, though similar in size, does not form intimate contacts with the galactose of αGalCer and, thus, αGalCer is presented more tilted at the opening of the boCD1d binding groove. Superposition of mouse, human and boCD1d presenting αGalCer reveals that boCD1d presents αGalCer more similar to human CD1d, thus likely explaining the slight cross-species activation of human NKT cells, however the binding chemistries between human and bovine CD1d are different. For human CD1d, the bulky trypophane side (Trp153) leads to the tilting of the galactose, while still maintaining the two important H bonds between Asp151 and the 2″ and 3″-OH of galactose [29], while bovine CD1d (as well as mouse CD1d) has a glycine residue at this position and the lack of forming the equivalen H bonds with Asn151 in boCD1d likely lead to the altered and tilted αGalCer presentation. The importance of the Asp151 sidechain has further been demonstrated by mutagenesis studies using mouse CD1d. Mutation of the equivalent Asp153 to asparagine leads to complete loss of iNKT cell activation further demonstrating why boCD1d is unable to activate mouse iNKT cells when presenting short-chain αGalCer. However, αGalCer itself is an unphysiological antigen, which mimics natural ligands for mouse and human iNKT cells. While mouse and human iNKT cells are evolutionary conserved and generally respond to the same antigens, this is not true for the more recently identified microbial antigens. Especially for *Borrelia burgdorferi* α-galactosyl diacylglycerol (αGalDAG) lipids, the precise nature of the diacylglycerol backbone determines antigenicity for human and mouse iNKT cells. Two αGalDAG species were identified that differ only in the fatty acid composition. While the antigen BbGL-2c (C_18∶1_/C_16∶0_) activates mouse iNKT cells but not human, BbGL-2f (C_18∶2_/C_18∶1_) activates only human iNKT cells [25], [35]. Therefore, it is possible that bovine pathogens differ in their lipid composition compared to human and mouse pathogens and as a result cattle have evolved lipid-reactive and boCD1d-restricted T cells that are likely different from human or mouse iNKT cells. This notion is further supported by the different size and shape of the A’ pocket of boCD1d, which allows for the binding of the majority of microbial diacylglycerolipids, which generally contain fatty acids with 16–18 carbons in length. In contrast, many glycosphingolipids, such as αGalCer, commonly incorporate long chain fatty acids ranging in length between 24 and 26 carbons, which would exceed the size of the boCD1d molecule. Therefore, boCD1d cannot present the same range of antigen as human and mouse CD1d can. The only other species for which CD1 crystal structures are available is chicken. Both chicken (ch)CD1-1 and chCD1-2 have been crystallized with endogenously bound lipids and the structures reveal two extremes in terms of size and shape of the hydrophobic binding groove, which dictates the type of lipids that can be presented by CD1 [36], [37]. While chCD1-2 has a primitive single pore suitable for binding of single chain lipids or fatty acids [37], chCD1-1 has an elaborate binding groove suitable to accommodate dual and possibly tri-acylated lipids [36]. Thus changes in size and shape of the binding groove likely reflect the encounter with different lipid structures from different pathogens during the course of CD1 evolution.

We further observed structural flexibility within the A’ pocket that can change the shape of the pocket. It is conceivable that this allows the accommodation of small acyl chain modifications, such as hydroxyl or methyl groups, which are found in different lipids or fatty acids such as tuberculostearic acid. Furthermore, lack of human T cell activation by bovine CD1 has also been seen for another bovine CD1 isotype, boCD1b3, which is unable to activate the human CD1b-restricted T cell line LDN5 when presenting glucose monomycolate [10], indicating that lipid reactive T cells in cattle differ from that of mouse and man.

## Supporting Information

Figure S1 **Antigen** **omit map electron density.** FoFc difference electron density omit maps were calculated before glycolipid fitting and are contoured at 3σ as a green mesh drawn around the headgroups of C_12∶0_-di-sufatide and C_16∶0_-αGalCer.
(TIF)
