# Supplementary figures and images for: Crystal Structures of Bovine CD1d Reveal Altered αGalCer Presentation and a Restricted A’ Pocket Unable to Bind Long-Chain Glycolipids
Source: PLoS One. 2012 Oct 23;7(10):e47989. doi: 10.1371/journal.pone.0047989 (PMC3479135; doi:10.1371/journal.pone.0047989)

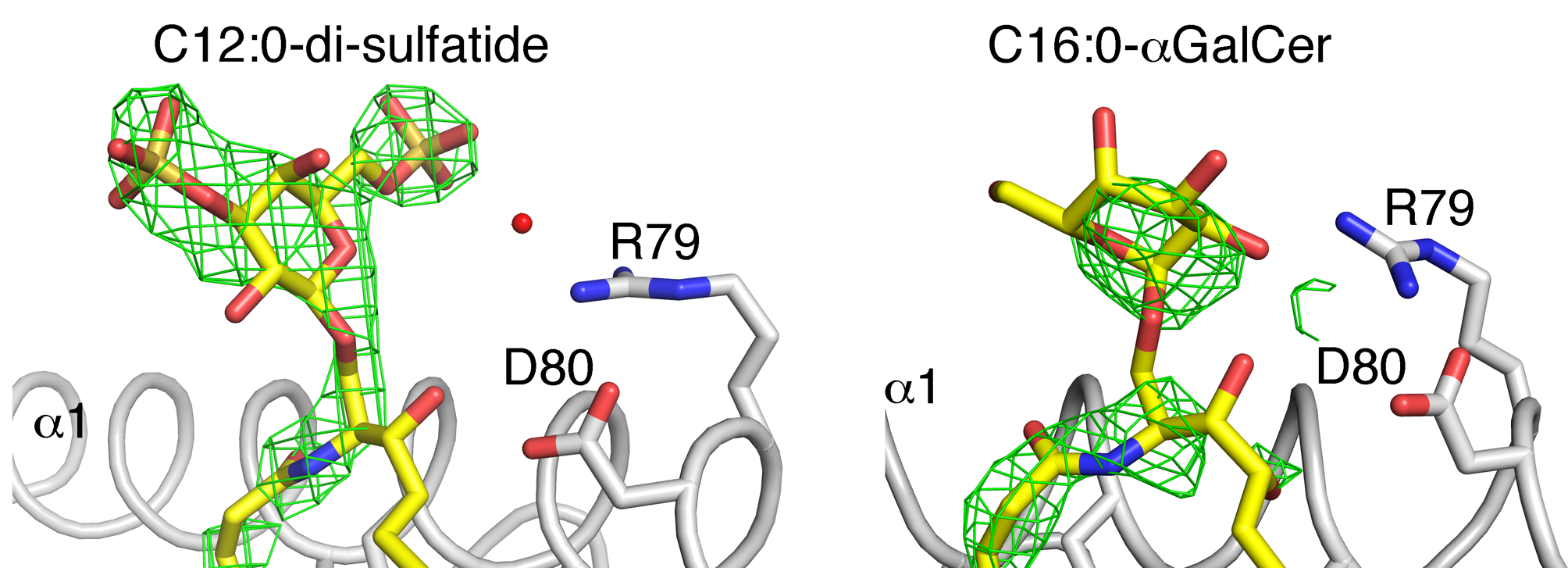

Supplement: Figure S1 — Antigen omit map electron density. FoFc difference electron density omit maps were calculated before glycolipid fitting and are contoured at 3σ as a green mesh drawn around the headgroups of C12∶0-di-sufatide and C16∶0-αGalCer. (TIF) [file pone.0047989.s001.tif]
